# Application of Cascaded TFBG for Wavelength-Shift-Based SRI Measurement with Reduced Polarization Cross-Sensitivity

**DOI:** 10.3390/s25061831

**Published:** 2025-03-15

**Authors:** Damian Harasim, Piotr Kisała

**Affiliations:** Faculty of Electrical Engineering and Computer Science, Lublin University of Technology, Nadbystrzycka Str. 38D, 20-618 Lublin, Poland; p.kisala@pollub.pl

**Keywords:** tilted Bragg grating, refractometry, light polarization, cascaded fiber structures

## Abstract

The wavelength shift in TFBG cladding resonance is a practical parameter due to its independence from power fluctuations coming from the light source or fiber bends. It is possible to select the cladding modes that are characterized by the center wavelength shifts while changing the refractive index of the surrounding medium. In the case of a single TFBG, this parameter is strongly dependent on the input light polarization angle. In this paper, we present the possibility of reducing interference of polarization changes to measure the refractive index by using the wavelength shift in Bragg grating cladding modes with the cascade structure called the perpendicular TFBG (P-TFBG). The wavelength shift in the selected cladding mode was analyzed and compared in the case of a single grating and proposed cascade. In the case of P-TFBG, the dependence on the corresponding cladding mode of a single TFBG with the same inscription parameters is reduced to 16.15%. The analysis of mode wavelength instead of the previously reported amplitude provides a wider measurement range of possible SRI and protects the results from power fluctuations.

## 1. Introduction

Optical fiber sensors are an important part of measuring systems that detect many physical and chemical quantities and can be used in advanced systems [1,2]. This is undoubtedly linked to the rapid development of optical technologies, including research on imaging and spectroscopy techniques [3,4] and advances in algorithms for extracting parameters from spectra [5]. Sensors inscribed as periodic structures in optical fibers have many advantages, such as miniaturization and multiplexing capability, which makes them increasingly popular. The development of miniaturized and stabilized light sources, such as tunable lasers and superluminescent diodes (SLEDs), allows for the construction of compact photonic measurement systems. The popularity of photonic tools is increasing, for example, in the field of medical applications in pathogen detection or refractive index measurement [6,7]. The periodic structures inscribed in the fiber core are very promising for methods of detecting many quantities, of which the refractive index of the surrounding medium is one of the most commonly used. The basic form of these photonic structures is called uniform fiber Bragg gratings (FBGs), which are inscribed by creating repeatable zones of increased refractive index in the fiber core. These zones are created as a series of fringes that are parallel to the fiber cross-sectional plane [8]. It is possible to introduce many modifications to uniform structures, such as various amplitudes of fiber core refractive index perturbations (apodization), creating a differential distance period between zones of increased RI and introducing a certain angle between created diffraction fringes and the fiber cross-section plane. The last modification resulted in the inscription of tilted fiber Bragg gratings (TFBGs) [9,10].

In the case of methods using fiber periodic structures, the polarization angle of the introduced light definitely affects the results of measurement. The TFBGs presented in this paper are a great example of structures with changes in spectral characteristics of the incident light polarization angle. The angle of tilt introduced to the diffraction fringes induces a dual coupling nature: the core Bragg mode, which propagates backwards, and a series of resonances coupled on the cladding–core boundary with mode center wavelengths shorter than the core Bragg mode. Sensors based on the periodic structures inscribed in the fiber core have great sensitivity to change in strain and temperature [11,12]. Contrary to the Bragg resonance, the spectra of cladding modes are modified with changes in the surrounding refractive index (SRI) of the medium in which the fiber is immersed due to the cladding–medium interface [13,14]. The intensity of the spectral changes in the selected modes is related to the effective refractive index and the distribution of its mode field. The effects described in this section are the source of changes in both the wavelength and the transmission of the cladding mode spectrum TFBGs, which are inscribed by tilting a plane of the modulation of the refractive index according to the plane of the cross-section of the optical fiber, which disturbs the cylindrical symmetry of the optical fiber. The unique characteristics of fiber sensors are also used to obtain the double functionality of the sensors, such as simultaneous measurement of curvature and strain [15] or temperature and strain [16]. In addition, selective sensitivity of TFBG-based sensors is important, especially in the field of biosensing [17,18].

Refractometers using TFBGs as transducers are a separate group of sensor systems [19]. Most of the SRI TFBG sensors previously presented are sensitive to changes in the polarization plane angle of the introduced light [20]. Due to this property, precise control of the input light polarization angle is required. In some other works, the authors proposed using the polarization controllers in an input fiber circuit to avoid fiber rotation to reduce the effect of induced birefringence caused by internal stress in the fiber. The above-mentioned solution makes all measurement systems more expensive and complicated. A sensor containing a grating cannot be replaced or even moved because of the possible changes between the geometry of the TFBG’s internal structure and the introduced light polarization. One of the presented approaches to achieving polarization insensitivity is to use global methods that are based on analyzing the broad wavelength part of the spectrum that contains many cladding resonances [21,22]. In all measurements it is necessary to use an optical spectrum analyzer.

In applications where small changes in SRI are measured, a survey can be carried out based on a wavelength shift or amplitude change in a single cladding mode [23]. In addition, the widely described methods of measuring the refractive index require precise control of the input of the light polarization angle.

In this research, we propose a measurement configuration that reduces the polarization cross-sensitivity of the TFBG cladding mode wavelength shift applied to an in-fiber refractometer setup. The measurement of the refractive index of the surrounding medium is carried out by the cladding mode, which shows a center wavelength shift in the assumed range of SRIs. The presented method is based on the inscription of a cascaded structure that contains two TFBGs with extremely matched wavelengths and reflectivities of the matched cladding resonances. The experimental results presented in the paper show that this approach is promising for obtaining reliability in the results of the measurement of the wavelength shift that is modulated with any angle of linear polarization of the induced light. The results presented in the previous paper [24] are based on the analysis of the transmission coefficients measured in selected wavelengths where the specific resonance reaches the minimum. Analysis of resonance wavelengths has many advantages over intensity measurements and results, with independence of changes in light intensity. This is important because the intensity of input light may be affected by fluctuations in the source power or by the curvature of the fibers used to transmit the optical signal. In this paper, it is also presented that the analysis of the wavelength changes in the selected cladding mode provides coverage of wider range of the surrounding medium refractive index than in the case of the transmission of the “cutoff” mode [24]. The comparison of the cross-sensitivity of SRI measurement based on the shift in wavelength from polarization changes presented in this work indicates the possibility of using a P-TFBG sensor and a conventional FBG filter to construct a measurement system. When a single TFBG is used, the dependence on polarization changes is a significant difficulty in the construction of real measuring systems. In addition, the results presented can be the basis for new analysis using numerical methods, which will increase the range and resolution of the measurements while remaining independent of polarization changes, unlike a single TFBG sensor.

## 2. Materials and Methods

### 2.1. Principles of the TFBG Operation

It was observed that the cladding modes excited in optical fibers with a diameter of 125 μm form two well-defined groups. The electric fields of the TE_0n_ and HE_mn_ vector modes are mainly characterized by S-type polarization related to the incidence plane of the fiber boundary. The second group of P-polarized modes TM_0n_ and EH_mn_ have electric fields parallel to the incidence plane [25]. In the case of the TFBG, two types of cladding mode families can be excited according to linear polarized light at the grating input [26]. The effective index of the cladding modes depends on the SRI because they are guided by the cladding–boundary interface. If the SRI is small, discrete resonances excited by core–cladding coupling exist in the short wavelength range of transmission. When an increase in the SRI occurs, high-order cladding modes are changed, first with a cutoff and then gradually transforming into radiation modes.

It is assumed that the light power input into the TFBG is *P_in_*, and the power of each P- and S-resonance is defined as:

*P_p_* = (1 − *α*)·*P_in_*; *P_S_* = *αP_in_*, (1)

where *α* is the ratio of the S-resonance, and *α* = 0 corresponds to an angle of 90° and *α* = 1 to an angle of 0°.

The spectrum of the TFBG depends on *α* and can be expressed as [27]:

(2) $$T\left(\lambda ,\;\alpha \right)=\alpha T_{S}\left(\lambda \right)+\left(1-\alpha \right)T_{P}\left(\lambda \right),$$

where *T_P_*(*λ*) and *T_S_*(*λ*) are the transmission spectra of the grating under P- and S-polarization excitation. S-polarization is perpendicular to the incidence plane of the fiber.

Phase-matching conditions can be used to determine wavelengths of the core mode (*λ_core_*) and the backward-propagating cladding modes(*λ_clad_*) [28]:

(3) $$\begin{array}{c} \lambda_{core}=2n_{eff}^{core}(\lambda_{core})\Lambda_{g},\\ \lambda_{clad}=\left[n_{eff}^{core}(\lambda_{clad})+n_{eff}^{clad}(\lambda_{clad})\right]\Lambda_{g}, \end{array}$$

where $n_{eff}^{core}(\lambda_{clad})$ and $n_{eff}^{core}(\lambda_{core})$ are the effective indexes of the core mode at *λ_clad_* and *λ_core_* wavelengths, respectively; $n_{eff}^{clad}(\lambda_{clad})$ is the effective refractive index of the cladding mode at wavelength *λ_clad_*; and *Λ_g_* is the period of grating along the axis of the fiber. Changes in the surrounding medium index maintain the same wavelength as the core mode *λ_core_*, while the *λ_clad_* cladding resonance wavelength is shifted [29]. As mentioned above, in order to consider the polarization effects in TFBG spectra, the fields are expanded by two orthogonally polarized resonances: P-mode and S-mode resonances. These two types of resonance cannot couple to each other.

### 2.2. The Polarization Effect on the SRI Measurement Using the Wavelemgth Shift in TFBG Cladding Modes

**Figure 1:**
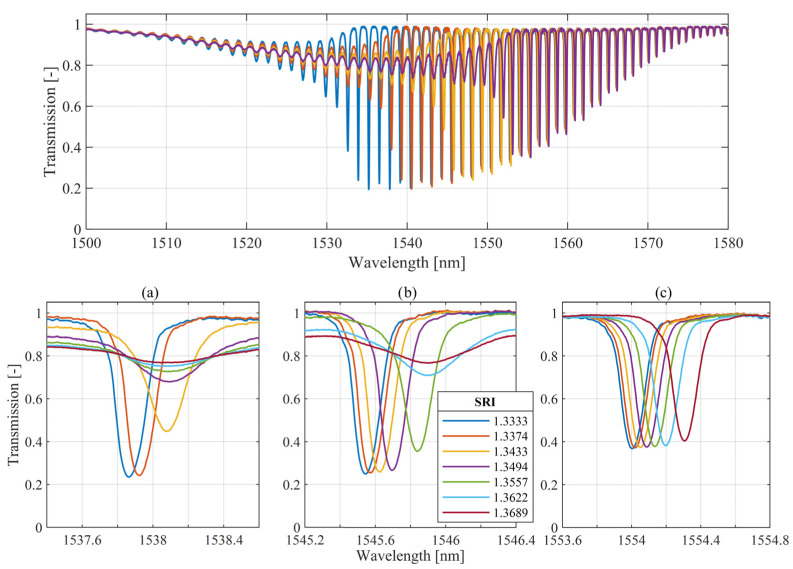
Comparison of the transmission spectral response of the 7° TFBG for various surrounding medium RI; insets (**a**–**c**) show detailed views of different cladding modes.

There are developed methods that use the transmission intensity of the selected cladding mode, which reacts with a strong coupling change to changes in the SRI in a specific range. The “cutoff” mode also presents a wavelength shift, related to the refractive index of the surrounding medium. The analysis of the TFBG spectra measured for TDBFs immersed in solutions with an increasing refractive index shows that the cladding modes observed for wavelengths longer than the cutoff mode react with the spectral shift. It is also visible that it is possible to select modes where the transmission coefficient shows small changes with SRI changes. Figure 1 shows the transmission spectra of the TFBG with an internal structure angle *Θ_TFBG_* equal to 7°, which is measured for gratings immersed in solutions with different refractive indexes. 

Figure 1a–c show different cladding modes from highest order (with the shortest wavelengths) to lowest order (with the longest wavelengths) respectively. Figure 1c presents the spectra of the cladding mode with a longer central wavelength, which react by shifting without strong changes in the transmission coefficient. It is also visible that the wavelength shift is nonlinear according to the SRI growth. Gratings used in the experiments were inscribed using the phase mask method in Ge-doped photosensitive fiber. The grating period was 550 nm.

**Figure 2:**
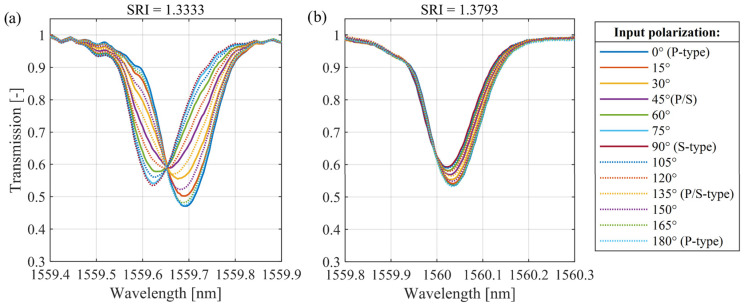
Spectral characteristics of the selected low-order cladding mode versus various input light polarization angles of the 7° TFBG immersed in (**a**) water RI = 1.33333 and (**b**) solution with RI = 1.3793.

The properties presented in Figure 1c of the lower-order cladding mode related to wavelength shift can be used in SRI sensing systems. However, the results shown in Figure 1 were obtained assuming the same stable state of the polarization angle of the input light. Light sources used in optical fiber technology generate radiation with a certain polarization state. It is an important direction for the development of fiber sensors to achieve structures that provide as much independence as possible from the state of polarization. Figure 2a shows the transmission spectra measured for various input light polarization angles of the same cladding mode as in the case of Figure 1b, with the TFBG sensor immersed in the solution with SRI = 1.3333. In both cases, changes in the polarization state have a strong influence on both the wavelength and the transmission of cladding mode spectra, while the refractive index of the surrounding medium is not changed.

The characteristics shown in Figure 2 show that an increase in SRI causes a high wavelength shift in the selected TFBG cladding mode and spectral changes due to the polarization dependence of the spectral parameters. In the case of SRIs near water (1.3333), when the sensitivity of the wavelength shift is lower, spectral changes caused by the rotation of the polarization plane can completely distort the measurements. In this work, we focus on spectral analysis of the cladding mode that responds to RI changes in the surrounding medium by a central wavelength shift. As has already been mentioned, this type of analysis based on wavelength tracking has a strong advantage over methods using transmission intensity. The spectral diagram shown in Figure 1 clearly shows that the wavelength demodulation of the selected cladding mode provides a wider range of possible RI measurements than the most widely described transmission analysis of the cutoff mode.

Figure 2a shows the transmission spectra of the selected TFBG mode with a 7° angle under variable light polarization angles when the sensor is immersed in water (RI = 1.3333). The excitation of the “even” mode with a longer wavelength is the result of the introduction of light with an S-type polarization angle. Changes in the light polarization angle cause a reduction in the even resonance intensity, resulting in an increase in the amplitude of the “odd” mode with a shorter wavelength. The intensity changes in each mode part can be used in the experimental configurations to measure rotation angle by analyzing specific spectral parameters [30]. In addition to the possibility of transmitting information about the angle of rotation, the polarization sensitivity causes serious disturbances in other physical quantity measurements. The spectra of the same cladding mode of the sensor immersed in the solution with RI = 1.3793 are shown in Figure 2b. It can be seen that the polarization dependence of the center wavelength is reduced but still creates significant disturbances in the measurement results. It can also be seen that the resonance wavelength reaches extreme values when the P-state changes to the S-state. Further rotation of the angle of the polarization plane leads to a return to the P-state. Therefore, in the following chapters, the analysis of the complete polarization rotation is abandoned, in favor of a 90-degree change from the P-state to the S-state.

This additional error caused by the changes in the input light polarization is unacceptable when refractive index measurements are carried out. The polarization of the input light should be controlled, which causes additional complications for the measurement system and requires the use of expensive optical components that must also be stabilized.

### 2.3. Comparison of Polarization Dependence of SRI Measurements Using TFBG and P-TFBG Cladding Mode Shift

One of the possible solutions to improve the insensitivity of the rotation of input light polarization during SRI measurement with a TFBG is the application of two orthogonally oriented TFBGs. As presented in [24], the appropriate orientation of two tilted gratings with perfectly matched spectra can be used to reduce undesirable spectral changes caused by uncontrolled changes in the polarization parameters of the input light caused, for example, by the movement of fibers used as a signal medium. In this section, we compare the spectral properties of a selected cladding mode of single TFBG with an internal angle of 7° and a cascaded P-TFBG structure in the wavelength-shift domain. The P-TFBG was created by the inscription of two TFBGs with the same internal angle of 7° and transmission coefficient. The second grating was inscribed in the fiber rotated by 90° around the central axis of the fiber.

**Figure 3:**
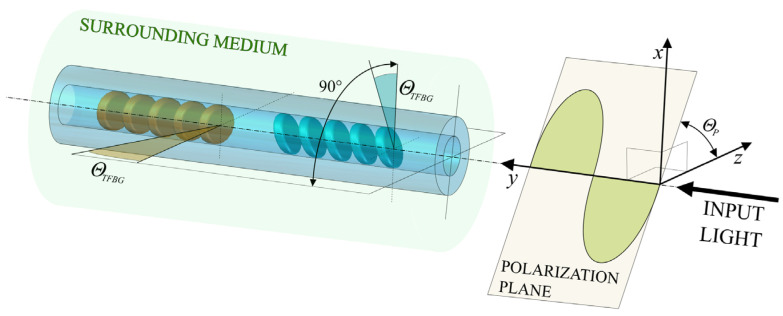
Schematic view of the P-TFBG structure, inscribed in the single mode fiber, with an indication of the polarization angle according to the geometry of the gratings.

As shown in Formula (1), the coupling between the two orthogonal resonance states depends on the *α* factor. In the case of the proposed P-TFBG structure, the effects of these changes will overlap. A schematic diagram of the P-TFBG structure immersed in the solution is presented in Figure 3. The angles of the planes of input light polarization are also marked.

Fiber Bragg gratings were inscribed by using the phase mask method using a 248 nm BraggStar Coherent (Saxonburg, PA, USA) excimer laser on a Ge-doped THORLABS GF1 fiber (Newton, NJ, USA). In order to understand the results of the present research, it is crucial that the TFBG spatial asymmetry create sensitivity to polarization changes. Thus, it is necessary to fix the polarization orientation of the light with respect to the geometry of the diffraction fringes of the gratings. The following results maintain the polarization orientation states (P, S) in the TFBG and P-TFBG, as shown in Figure 3.

**Figure 4:**
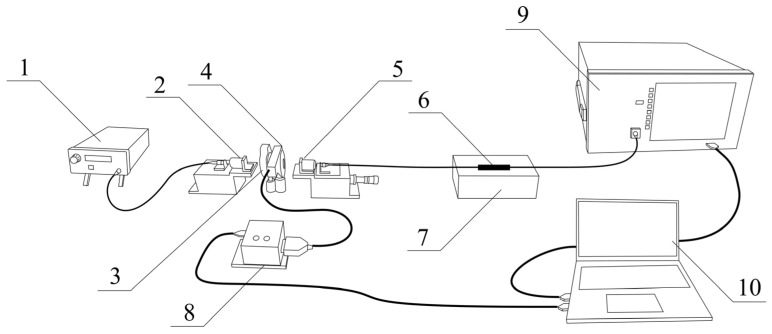
Schematic view of the experimental configuration used for the TFBG and P-TFBG spectra measurements, with grating sensors immersed in media with a variable SRI.

The results presented in this paper were obtained from measurements conducted using laboratory class equipment, which is schematically presented in Figure 4. The light source was a broadband SLED S5FC1005P THORLABS (Newton, NJ, USA), and all spectra were measured with an Optical Spectrum Analyzer (OSA) Yokogawa AQ6370D, Tokyo, Japan with a 0.02 nm resolution. The components of the experimental setup are as follows: 1—SLED source, 2—collimator, 3—polarizer, 4—half-wave plate in a motorized rotation mount, 5—collimator, 6—TFBG/P-TFBG immersed in solution, 7—container with solution, 8—driver of the motorized rotation mount with a half-wave plate, 9—OSA, and 10—PC computer with specialized software.

The liquids with different refractive indexes were prepared as glucose solutions with known proportions. The reference RI measurement of the solutions used was carried out using a digital refractometer Atago RX-5000i-Plus, Conbest, Krakow, Poland with a resolution of 0.00002 RIU. The state of the input light polarization was determined by using a polarizer (to obtain a high degree of polarization) and a half-wave plate, which was used to obtain the desired angle of the polarization plane. Because the two TFBGs that create the P-TFBG cascade have the same reflection coefficient for every cladding mode, the reflectivity of each resonance is greater for these structures than for the TFBG because the light is transmitted through both gratings. The angle of the input polarization plane angle was set using a motorized rotation mount driven by a computer with dedicated software. Comparison between the selected cladding mode of the P-TFBG and the single TFBG shows that there is no significant increase in sensitivity to wavelength change in the surrounding refractive index.

**Figure 5:**
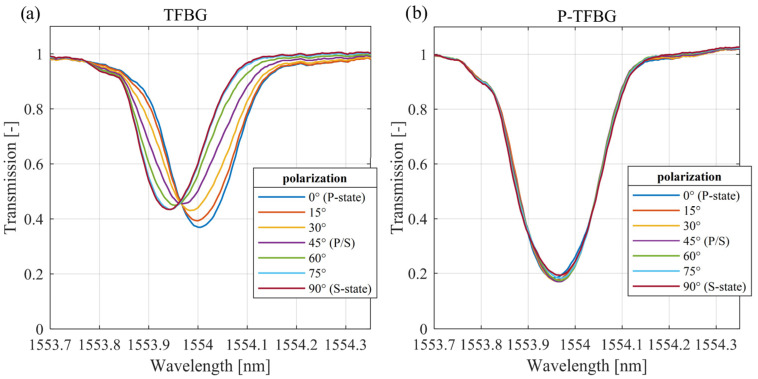
The transmission spectra of the 7° TFBG (inset (**a**)) and P-TFBG cascade (inset (**b**)) measured for the different input light polarization angles of sensors immersed in water.

The main research objective of this paper is to compare the wavelength shift of a corresponding cladding mode of a single TFBG and cascaded P-TFBG gratings at different introduced light polarization plane angles. The detailed analysis provided in this section applies to the cladding mode shown in Figure 1c. This resonance was selected according to the observable wavelength shift, with simultaneous insensitivity of the reflectivity when the SRI changes. The polarization effects on the spectra of the cladding modes that were analyzed during SRI measurements are shown in Figure 5. In this figure, we show the transmission spectra of the selected cladding mode of the 7-degree TFBG and P-TFBG, arranged as shown in Figure 3. Spectra were measured for the sensor placed in a single SRI equal to 1.3333 and various input light polarization angles. The figure shows the characteristics measured for water (RI = 1.3333) because, as shown earlier in Figure 2, the sensitivity of the spectrum to polarization changes is the greatest at the lowest SRI value in the selected range.

A comparison of the plots presented in Figure 5a,b shows that the polarization of input light has less influence in the case of the P-TFBG design with the cascaded gratings. Changes observed in the spectra of a single TFBG sensor are caused by the rotation of the input light polarization plane.

According to the data shown in Figure 5b, in case of the P-TFBG, the wavelength shift that occurs for changes in the SRI can be considered insensitive to changes in the input light polarization. The shift in the center wavelength of the selected cladding mode and the transmission change are minimized. The usefulness of the proposed sensor scheme is proven by the comparison of the transmission spectra shown.

## 3. Results and Discussion

**Figure 6:**
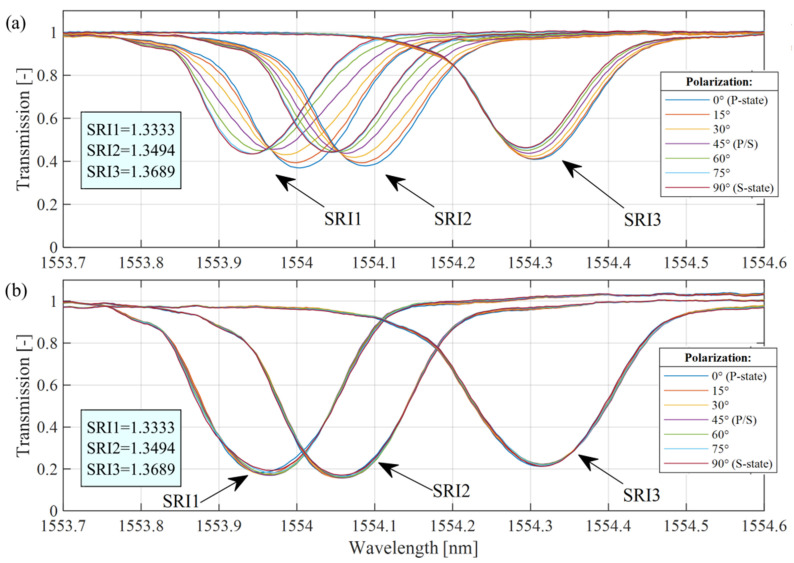
Direct comparison of the selected cladding mode transmission spectra of (**a**) 7° TFBG and (**b**) the proposed P-TFBG cascade, measured for various angles of the input light polarization angle and three values of the SRI: 1.3333, 1.3494, and 1.3689.

It is important to show how this gain in the polarization insensitivity relates to changes in the SRI in a wider range and at more measurement points. It is shown that the differences between wavelength changes varies depending on changes in the SRI for the variable angle of the input light polarization angle. To present the advantages of the proposed cascaded sensor, the characteristics of the spectra of a single 7° TFBG and a cascaded P-TFBG were obtained. Figure 6 shows the spectra of the TFBG and P-TFBG measured for fibers immersed in three solutions with SRIs of 1.3333, 1.3494, and 1.3689. For each SRI, the input light polarization angle changed from 0° (P) to 90° (S), with a 15° step.

The plots shown in Figure 6 show that for the cladding modes with wavelengths longer than the “cutoff” mode, the sensitivity of the spectral shift is greater for the higher values of the surrounding medium RI. Table 1 and Table 2 show the differences between the average value of wavelength determined from the cladding mode for different polarizations and the specific value for each of the polarizations. The results presented in the tables were calculated based on the measured spectra presented in Figure 6a (TFBG) and Figure 6b (P-TFBG).

**Figure 7:**
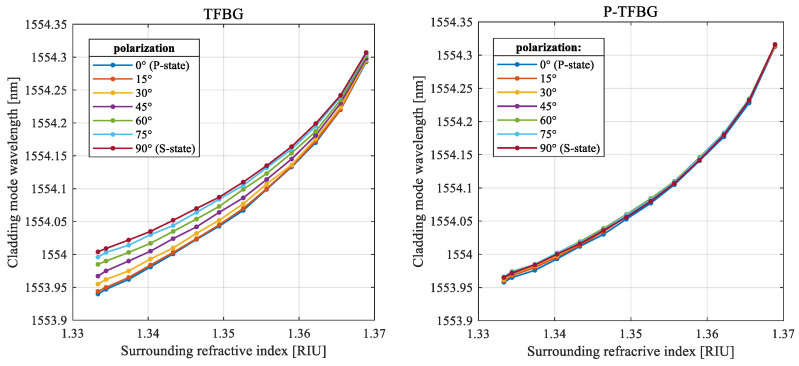
Characteristics of the selected cladding mode center wavelength measured for increasing the value of the surrounding medium RI in 7 cases of input light polarization angles.

Analyzing the numerical values presented in the tables shows that the differences between extreme values are approximately ten times smaller in the case of the cascaded P-TFBG compared to the conventional TFBG. The dependence on the polarization plane angle decreases, while the SRI grows. Figure 7 contains two sets of characteristics that present the center wavelength shift in the cladding mode in the medium with various RIs and changing input light polarization angles for two compared structures: (a) 7° TFBG and (b) the proposed cascaded P-TFBG. The wavelength of the cladding mode grows monotonically when the angle of the polarization changes from the P- to the S-state.

The average wavelength shifts in the cladding mode caused by the opposite cases of input light polarization are 0.04285 nm for the TFBG and 0.00692 nm for the P-TFBG. As a result, the average dependence of the wavelength of the selected cladding mode on the polarization of the input light is reduced to 16.15% of the initial value obtained for a conventional TFBG grating.

**Figure 8:**
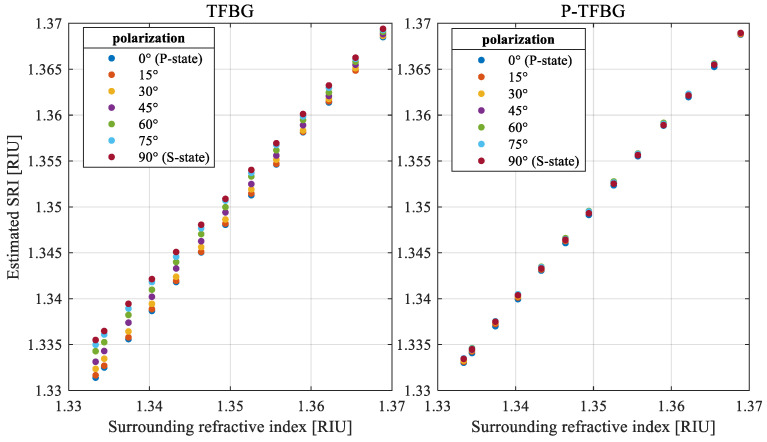
Characteristics of the measured SRI value spread caused by various input polarization angles over the selected range of SRI for the TFBG and P-TFBG design.

It is also valuable to present how changes in the polarization plane angle input affect SRI measurements based on the wavelength shift in the cladding mode. The spread of wavelengths of the selected mode designated for the known SRI and various angles of the polarization plane is presented in Figure 8. The presented results prove that the influence of the polarization on the central wavelength of the cladding mode is stronger for lower SRI values. It can be observed that the differences in the SRI measured over various input polarization plane angles are significantly lower for the P-TFBG.

**Figure 9:**
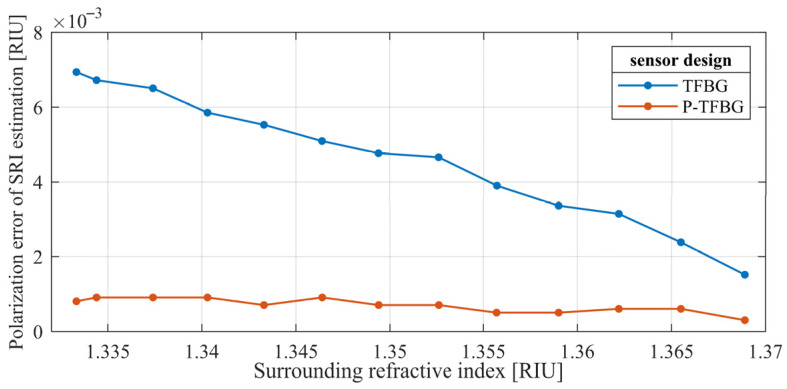
The estimated error value of SRI demodulation for a single TFBG sensor and the proposed P-TFBG with the same technical parameters.

The comparison of the characteristics shown in Figure 8 shows that the error in estimation of the SRI using wavelength shift is lower for the P-TFBG compared to the TFBG with the same tilt angle. Figure 9 provides a direct comparison of the SRI error caused by changes in input light polarization of the 7° TFBG and the P-TFBG at the same tilt.

The estimated error value for the TFBG is reduced, while the SRI is increased, which is related to the behavior of the selected cladding mode. For higher values of RI of the medium, the cladding mode behaves as a “cutoff” mode, and instead of a wavelength shift, it starts to reduce the refection. This research is based on measuring the SRI in the range of wavelength shift in the cladding mode without changes in the transmission amplitude. It can also be seen that the error value calculated for the P-TFBG is similar over the assumed RI range. It is beneficial to create complex sensing systems.

In general, increases in the SRI results in subsequent cladding resonances to penetrate the mode field into the surrounding medium. For this reason, the properties described will actually repeat for the subsequent modes with longer wavelengths, as the SRI is further increased. It can therefore be assumed that the analysis of the wavelength of several cladding modes would allow the measurement range to be expanded while maintaining a limited cross-sensitivity to polarization changes.

## 4. Conclusions

The possibility of using P-TFBG grating, which is created by a cascade of two TFBGs, for measuring the SRI with reduced polarization cross-sensitivity is presented. Polarization is one of the basic parameters that describes the light introduced into periodic fiber structures. The coupling properties and spectral response of excited TFBG cladding modes are strongly affected by the input light polarization angle. As presented in this paper, a series of cladding modes react with a change in the transmission coefficient and center wavelength when a fiber with an inscribed grating is immersed in a water solution. For selected ranges of the surrounding medium RI, it is possible to select cladding modes in which the wavelength changes monotonically and, at the same time, the transmission coefficient is not affected. For optical fiber measurements, it is difficult to estimate the angle of input light polarization directly before the TFBG. This requires controlling polarization states, which causes complications in the optical circuit. Using a 7° TFBG as an example, we present wavelength shift range caused by the changes in the angle of the introduced light polarization plane. The proposed method is based on the application of two cascaded TFBGs with an internal tilt of 7°. The geometry of the internal structure of the first TFBG is rotated 90° in relation to the second grating. The placement of two TFBGs in the proposed cascaded geometry significantly reduces the adverse effects of wavelength shifting in the cladding modes of both gratings. This is because the response of the spectrum of the selected cladding mode has the opposite effect after crossing a certain plane between the geometry of the internal TFBG structure and the introduced angle of the light plane. This paper presents an analysis of the change in the selected cladding mode wavelength caused by changes in the SRI from 1.3333 to 1.3689 and the rotation of the light polarization angle of the input. The average difference in the cladding mode wavelength of a single TFBG changes by 0.03553 nm for extreme polarization states (between the P-state and the S-state). For the cascaded P-TFBG structure, this wavelength shift amounts to 0.00487 nm. Consequently, in single TFBGs, with the same 7° tilt angle, the P-TFBG application reduces the undesirable shift caused by changes in the input polarization angle to 16.15%. The achievement of a periodic structure that reduces cross-sensitivity to changes in the light polarization of the input is important for reducing the cost and complexity of the sensor system. If the cross-sensitivity of the P-TFBG is sufficiently reduced, it is not necessary to precisely control the polarization state of the light introduced into the cascade structure. The possibility of replacing of such a sensor will result in there being no need to recalibrate the orientation between the P-TFBG internal geometry and the introduced light polarization plane angle. The analysis of the resonance, which only reacts with wavelength shift and without a change in the reflectivity, allows for the use of dedicated interrogation systems, which are developed to detect shifts in center wavelengths of FBG sensors.

## Figures and Tables

**Table 1:** Changes in the wavelength of the selected TFBG cladding mode for different polarization angles of the input light.

SRI	Mean Wavelength	Difference Between Actual Wavelength and Mean Value						
		Input Light Polarization Angle [°]						
		0	15	30	45	60	75	90
1.3333	1553.9701	−0.0301	−0.0261	−0.0151	−0.0031	0.0149	0.0259	0.0339
1.3374	1553.9901	−0.0281	−0.0251	−0.0151	−0.0001	0.0129	0.0239	0.0319
1.3433	1554.0241	−0.0231	−0.0211	−0.0141	−0.0001	0.0109	0.0199	0.0279
1.3494	1554.0640	−0.0210	−0.0190	−0.0120	0.0000	0.0090	0.0200	0.0230
1.3557	1554.1157	−0.0167	−0.0157	−0.0087	−0.0017	0.0073	0.0163	0.0193
1.3622	1554.1828	−0.0129	−0.0109	−0.0069	−0.0019	0.0041	0.0121	0.0161
1.3689	1554.2993	−0.0063	−0.0043	−0.0043	−0.0013	0.0027	0.0057	0.0077

**Table 2:** Changes in the wavelength of the selected P-TFBG cladding mode for different polarization angles of the input light.

SRI	Mean Wavelength	Difference Between Actual Wavelength and Mean Value						
		Input Light Polarization Angle [°]						
		0	15	30	45	60	75	90
1.3333	1553.963	−0.0051	−0.0031	−0.0011	0.0019	0.0029	0.0029	0.0019
1.3374	1553.983	−0.0066	−0.0026	0.0004	0.0024	0.0024	0.0024	0.0014
1.3433	1554.016	−0.0040	−0.0020	0.0000	0.0010	0.0030	0.0020	0.0000
1.3494	1554.058	−0.0046	−0.0006	0.0004	0.0024	0.0024	0.0014	−0.0016
1.3557	1554.108	−0.0030	−0.0010	0.0000	0.0020	0.0020	0.0010	−0.0010
1.3622	1554.181	−0.0040	−0.0010	0.0000	0.0020	0.0020	0.0020	−0.0010
1.3689	1554.315	−0.0021	−0.0011	−0.0001	0.0009	0.0009	0.0009	0.0009

## Data Availability

Data are available on request due to restrictions.
